# *Clostridium difficile* 027-associated pseudomembranous colitis after short-term treatment with cefuroxime and cephalexin in an elderly orthopedic patient: a case report

**DOI:** 10.1186/1756-0500-5-609

**Published:** 2012-10-31

**Authors:** Kirstine Kobberøe Søgaard, Tove Ejlertsen, Henrik Carl Schønheyder

**Affiliations:** 1 Department of Clinical Microbiology, Aalborg Hospital, Aarhus University Hospital, Mølleparkvej 10 6 Sal, Aalborg DK- 9000, Denmark

**Keywords:** *Clostridium difficile*, Ribotype 027, Pseudomembranous colitis, Cefuroxime, Cephalexin

## Abstract

**Background:**

*Clostridium difficile* ribotype 027 has become increasingly prevalent in European countries. The clinical picture varies from self-limiting diarrhea to pseudomembranous colitis with toxic megacolon and ultimately death. Use of antibiotics is the principal risk factor; others include comorbidity, advanced age and hospitalization. However even with extensive knowledge of risk factors, it remains difficult to define “minimum risk,” as illustrated by the following case.

**Case presentation:**

An 80-year-old Danish man in good health was hospitalized for a penetrating knee injury. He received 5 days of intravenous cefuroxime after surgical revision and was discharged with oral cephalexin. Post-discharge he suffered from abdominal discomfort and was readmitted with ileus 4 days after discharge, i.e. 10 days after initiation of antibiotic treatment. His condition deteriorated, and pseudomembranous colitis was diagnosed. Due to lack of response to vancomycin and metronidazole, a total colectomy was performed. Stool cultures were positive for CD 027.

**Conclusion:**

Short-term use of cephalosporins may have induced CD 027 infection, and the patient’s age was the only identifiable risk factor for the fulminant course. Thus, even short-term prophylactic treatment with cephalosporins cannot be considered entirely safe.

## Background

The increasing incidence and severity of *Clostridium difficile* (CD) infections is becoming a major clinical concern impacting public health. The clinical picture varies from self-limiting diarrhea to pseudomembranous colitis with toxic megacolon and ultimately death. CD is an important cause of hospital-acquired as well as healthcare-associated disease [1]; furthermore, the incidence of community-associated disease tends to reach levels equal to hospital-acquired disease [2]. In Denmark, colitis caused by CD infection has increased markedly among hospitalized patients over a 10 year period [3], and since 2009 when registering of subtypes began, the number of infections with ribotype 027 has doubled [4]. In a European laboratory survey of CD patients, the proportion of ribotype 027 was 5% [5].

Previous use of antibiotics is the most important risk factor for CD infection [6], particularly the use of cephalosporins [7] and flouroquinolones [8]. The risk is highest during or within the first month after antibiotic use [9]. Other established risk factors include comorbidity, advanced age and immunosuppression [5]. CD infection can also occur among “low-risk” populations (i.e., young people, people without underlying illness, people not exposed to hospitals or antimicrobials) [2], but people in good health are generally not affected by serious CD infection. Those with serious CD infection often have a high degree of comorbidity and long exposure to antibiotics [10]. Only a few case reports indicate that grave CD 027 infections can also occur in patients without significant comorbidity [11,12]. To emphasize the risk of fulminant pseudomembranous colitis (PMC) associated with CD 027, we report a case in a patient with modest comorbidity given short-term cephalosporin treatment.

## Case presentation

**Figure 1:**
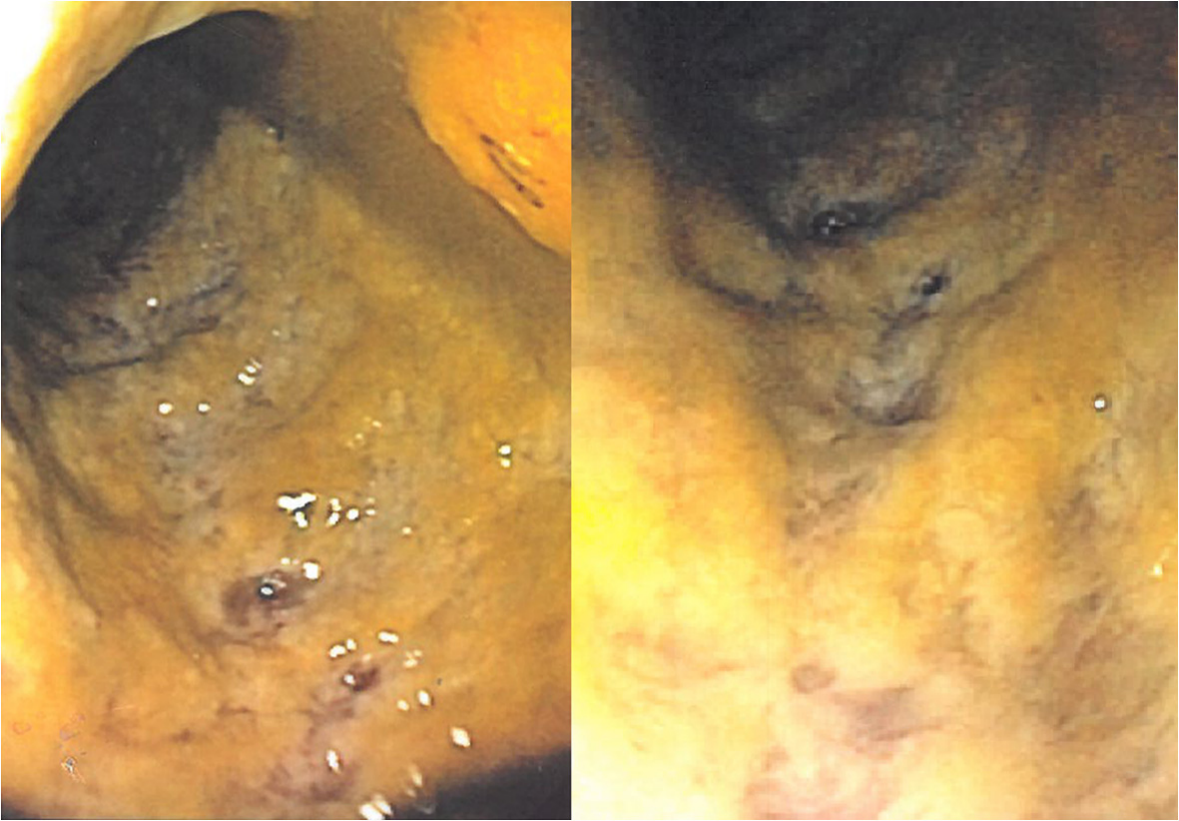
The endoscopic examination revealed an intestinal mucosa covered by adherent yellow-white plaques forming pseudomembranes.

An 80-year-old Danish man was admitted to hospital because of a 2-day history of increasing abdominal discomfort and constipation. Ten days earlier he had been hospitalized and treated for a penetrating knee injury, and had received a 5-day course of intravenous cefuroxime. Baseline comorbidity consisted of essential hypertension, mild sequelae after a minor stroke, and prostate cancer without proven dissemination. Previous surgical procedures were limited to appendectomy for perforated appendicitis 3 years earlier. The patient’s regular medications were bicalutamide, solifenacin, tamsulosin, simvastatin, losartan and clopidogrel, medications not known to be related to *Clostridium difficile* infection. While walking in the countryside, he had been hit by a small piece of metal from an agricultural machine. The object was removed surgically from the knee joint, and a ruptured collateral ligament was resutured. He was treated prophylactically with IV dicloxacillin 2 g at admission and IV cefuroxime 1.5 g during operation, followed by 750 mg q.i.d. for 5 days postoperatively. He was discharged on day 6 with oral cephalexin 1 g t.i.d (5 weeks’ treatment was scheduled). At the time of readmission (day 10), he was constipated and suffered from increasing abdominal discomfort, but he was clinically stabile, body temperature 36.9°C, serum C-reactive protein 296 mg/L (<10 mg/L), and white blood cell count 10.6 *10^9^/L (<10.0 *10^9^/L). However, his condition deteriorated rapidly to severe sepsis with high fever, hypotension and renal failure, and he was transferred to the intensive care unit. A CT scan of the abdomen revealed colitis, and sigmoidoscopy showed PMC affecting the distal colon up to the left colic flexure (Figure 1). Treatment with oral vancomycin and metronidazole was commenced, but repeated CT scans showed progression to the transverse and ascending colon. Four days after initiation of vancomycin and metronidazole (day 14 after surgery and initiation of cephalosporin treatment), a total colectomy was performed that left the patient with a permanent ileostomy. Stool culture was positive for CD 027, with genes for toxin A, toxin B and binary toxin.

## Methods

Stool samples were cultured on ChromID *C. difficile* agar (bioMérieux, Marcy l’Etoil, France) in an atmosphere composed of 86% N_2_, 7% H_2_ and 7% CO_2_ at 37°C for 48 hours. Isolates of *C. difficile* were characterized by PCR ribotyping, toxin gene profiling, and deletion studies undertaken by the National Reference Laboratory for Enteropathogenic Bacteria at Statens Serum Institut, Copenhagen, Denmark.

## Discussion

The majority of patients infected with CD 027 will suffer mild to severe diarrhea, whereas a few cases will proceed to PMC with toxic megacolon, sepsis and a possible fatal outcome. Cephalosporin use is associated with an increased risk of CD infection, and 3^rd^ generation cephalosporins are considered to have the highest impact on intestinal bacterial flora because an active metabolite is secreted into small intestine via the gallbladder [9]. However, a growing body of evidence shows that also 1^st^ and 2^nd^ generation cephalosporins (i.e. cephalexin and cefuroxime) increase the risk of CD infection [13-15]. Cefuroxime is, however, by some, considered to be safe when used prophylactically or therapeutically in orthopedic surgery [16,17]. Characterization of the risk factors for fulminant manifestation of the disease is sparse. A prospective study including 1,008 Canadian patients with CD infection found that age was associated with a severe outcome when the infection was due to CD 027 [18]. A retrospective cohort study of 2,334 hospitalized patients with CD colitis in the United States found immunosuppression and recent comprehensive surgical procedures to be common predisposing conditions for colectomy; yet none of the patients undergoing colectomy had had orthopedic surgery [19]. On this basis, we cautiously conclude that the short-term use of cephalosporins induced CD 027 infection in our patient, and that his advanced age was the main risk factor for the fulminant progression.

## Conclusion

We present a case of fulminant PMC associated with CD 027 in an elderly patient with only modest comorbidity who received a short course of dicloxacillin, cefuroxime, and cephalexin subsequent to a surgical revision of a knee injury. Apart from advanced age there was no underlying condition that explained the grave outcome in this patient. This finding further supports the overall principal of antimicrobial stewardship, which specifies to reduce inappropriate and excessive use of prophylactic cefuroxime and cephalexin.

## Consent

Written informed consent was obtained from the patient for publication of this case report. A copy of the written consent is available for review by the Editor-in-Chief of this journal.

## Abbreviations

CD: Clostridium Difficile; PMC: PseudoMembranous Colitis; q.i.d.: quater in die (4 times a day); t.i.d.: ter in die (3 times a day).

## Competing interests

The authors declare that they have no competing interests, and that they received no funding.

## Authors' contributions

KKS drafted the manuscript. HCS and TEJ critically revised the manuscript. All authors read and approved the final manuscript.
